# Development and validation of the Self-Regulation of Eating Behaviour Questionnaire for adults

**DOI:** 10.1186/s12966-016-0414-6

**Published:** 2016-08-02

**Authors:** Nathalie Kliemann, Rebecca J. Beeken, Jane Wardle, Fiona Johnson

**Affiliations:** Health Behaviour Research Centre, Department of Epidemiology and Public Health, University College London, Gower Street, London, WC1E 6BT UK

**Keywords:** Measurement, Questionnaire, Self-regulation, Self-control, Eating behaviour, Validity, Reliability

## Abstract

**Background:**

Eating self-regulatory capacity can help individuals to cope with the obesogenic environment and achieve, as well as maintain, a healthy weight and diet. At present, there is no comprehensive, reliable and valid questionnaire for assessing this capacity and measuring change in response to self-regulation interventions in adults. This paper reports the development of the Self-regulation of Eating Behaviour Questionnaire (SREBQ) for use in UK adults, and presents evidence for its reliability and construct validity.

The development of the SREBQ involved generation of an item pool, followed by two pilot studies (Samples 1 and 2) and a test of the questionnaire’s underlying factor structure (Sample 3). The final version of the SREBQ was then assessed for reliability and construct validity (Sample 4).

**Results:**

Development of the SREBQ resulted in a 5-item questionnaire. The face validity was satisfactory, as assessed by the pilot studies. The factor structure analysis (Sample 3) suggested that it has a single underlying factor, which was confirmed in a second sample (Sample 4). The SREBQ had strong construct validity, showing a positive correlation with general measures of self-regulation. It was also positively correlated with motivation and behavioural automaticity, and negatively correlated with food responsiveness and emotional over-eating (p < 0.001). It showed good discriminant validity, as it was only weakly associated with satiety responsiveness, food fussiness and slowness in eating.

**Conclusions:**

The SREBQ is a reliable and valid measure for assessment of eating self-regulatory capacity in the general UK adult population.

**Electronic supplementary material:**

The online version of this article (doi:10.1186/s12966-016-0414-6) contains supplementary material, which is available to authorized users.

## Background

Changes in dietary and physical activity patterns, largely attributable to environmental changes, promote a positive energy balance in many populations [1]. However, environmental cues to eat do not affect all people similarly and there is a need to understand individual-level factors that determine vulnerability to the development of obesity [2]. In recent years, it has been suggested that the capacity to self-regulate eating behaviours may moderate individual susceptibility to the obesogenic environment and support the maintenance of a healthy weight and diet [3, 4]. Behavioural self-regulation is likely to be a relatively stable construct [5], but one that can be improved through practice [3, 6]. As a consequence, interventions promoting self-regulation training may have the potential to support successful weight control [7] and the formation of healthy dietary habits [8]. In order to test this and to determine the effectiveness of interventions it is imperative to have a valid and reliable measure of eating self-regulatory capacity. However, at present no established and standardized self-report measures exist to assess eating self-regulatory skills in the adult population. The aim of this study was to address this gap by developing and validating a measure of eating self-regulatory capacity for adults.

### Defining self-regulation of eating behaviours

Self-regulation refers broadly to the multiple processes involved in goal-directed behaviour [9] and encompasses management of behaviour, thoughts, feelings, attention and environment in the pursuit of personal goals [10–13]. The ability to self-regulate can be applied to a range of behavioural domains [14] including eating behaviour. Studies have suggested that the capacity to self-regulate eating behaviours bridges the intention-behaviour gap [15, 16].

The specific mechanisms by which self-regulation operates and its principles vary according to different theoretical models. However, most explain self-regulation as a process (reflective and/or automatic) involving: setting goals and reference points; self-monitoring; appraising progress; and making adjustments when necessary or giving up [17–19]. Goal-setting is a prerequisite to regulating behaviour as the goal serves as a reference value [11]. The process of monitoring eating behaviour and comparing it to personal goals has been consistently associated with effective long-term goal pursuit [17, 20, 21]. Similarly, the process of forming coping and action plans to adjust behaviour when a discrepancy between behaviour and goal is noticed, has been linked to an increased likelihood of attaining the desired eating goal in both intervention [22] and longitudinal [15] studies. The ability to keep eating goals in mind has also proven to be an important eating self-regulatory skill that helps people to resist food temptations and achieve their eating intentions [23, 24]. Success in regulating eating behaviour is also dependent on sufficient capacity to achieve this in light of obstacles and temptations [6, 25] in the short- and long-term context [13].

There is substantial debate as to whether the capacity to self-regulate is limited [14]. An influential theory of self-control has suggested that self-regulation relies on a limited resource to operate, similar to energy or strength [26]. When these resources are exhausted, as a result of prior engagement in self-control effort, people become temporarily vulnerable to self-regulatory failure in the subsequent self-control attempt: so-called ‘Ego depletion’ [27]. However, this widely held belief has been challenged in a meta-analysis [28] and there is evidence that applying self-control over time in a consistent context may lead to more efficient and automatic self-regulation, and increase resistance to self-regulatory failure [9, 29]. Bargh and Williams [30] have reasoned that self-regulation actions tend to be conscious at the beginning and become automatic and less effortful over time.

### Assessment of self-regulation of eating behaviours

A number of scales have been designed to assess general self-regulatory skills. However, existing questionnaires do not focus directly on the self-management of eating behaviour [13, 19, 31–33]. Self-regulation of eating is likely to interact with biologically-mediated variation in appetite, and as a consequence, general self-regulation questionnaires show only modest associations with healthy eating behaviours and weight control [32–35]. A recently published questionnaire, the Tempest Self-Regulation Questionnaire for Eating (TESQ-E), has addressed this gap [12]. However, this new measure was specifically designed to assess adolescents’ eating self-regulation strategies for healthy eating. It does not address some of the main self-regulatory skills components, such as self-monitoring, appraising progress and reviewing and amending goals, and is not suitable for use with adults.

Additionally, some psychometric scales assessing eating behaviours have items that measure self-regulation components, but none assess self-regulation of eating behaviour uniquely and comprehensively. For example, the construct of dietary restraint [3, 36] overlaps with self-regulation, but restraint scales also assess a range of personality traits and eating tendencies (such as susceptibility to overeat and weight fluctuation, self-efficacy, appetitive traits and food choices) [37, 38]. Correlations between measures of dietary restraint and dietary intake are generally weak, and the presence of multiple constructs in restraint scales may account for the inconsistent results published over the past 40 years on the relationship between cognitive control and weight [3, 37, 38]. Scales assessing dietary restraint also assume a goal of weight loss, which may not always be central to dietary intentions [12, 23, 39–42].

### The present study

Given the lack of a comprehensive, reliable and valid questionnaire to assess eating self-regulatory capacity in adults, this paper reports the development of the Self-Regulation of Eating Behaviour Questionnaire (SREBQ) for adults. As goals are a prerequisite to applying self-regulation [11], the relevance of the SREBQ is limited to individuals who have an intention to either have a healthy diet or to not eat too much of foods they find tempting. Hence, the SREBQ measures self-regulatory capacity relative to eating intentions already established by the individual. It should be also clear that the SREBQ does not aim to assess each of the individual components involved in the process of self-regulation in isolation, nor what people do to control their eating. The purpose of the SREBQ is to assess how capable someone is at regulating their eating, and it takes into account the skills needed to successfully self-regulate healthy eating behaviour. We also present evidence for the reliability and construct validity of the SREBQ.

## Methods

**Fig. 1:**
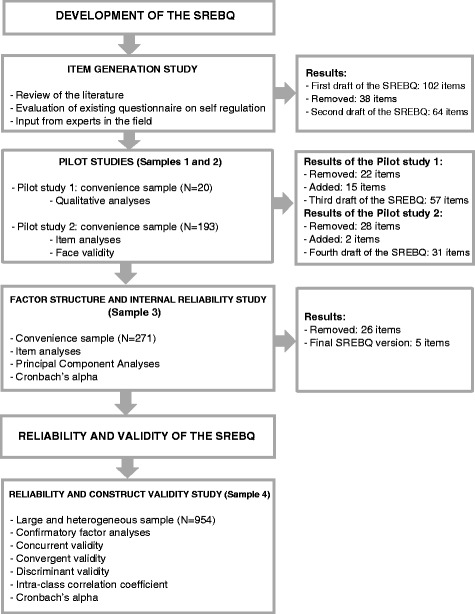
Flow diagram of the development and validation of the Self-regulation of Eating Behaviour Questionnaire (SREBQ)

The development of the SREBQ involved an item pool generation, followed by two pilot studies and a study exploring the questionnaire’s underlying factor structure and internal reliability. The results for the piloting, and the factor structure and internal reliability study are presented in the methods section, as they were part of the process of development of the SREBQ. The final version of the SREBQ was then administered to a different sample and had its reliability and construct validity assessed, as shown in Fig. 1.

### Development of the SREBQ

#### Item generation study

The aim of the SREBQ was to assess the capacity to self-regulate eating behaviour. Items were generated based on 1) A review of the literature on self-regulation of eating behaviour theory; 2) Existing questionnaires on self-regulation; and 3) Input from experts in the field. Criteria for inclusion of items in the item pool was that items should assess one of the key components of self-regulation (setting goals, self-monitoring, appraising progress, adjustments, overcoming barriers and giving up) and/or address the main capacities of self-regulation (behaviour, attention, affective and cognitive control). An initial large pool of 102 items was generated. Positively and negatively worded items were included to avoid ‘response bias’. The response scale format chosen for the questionnaire was a 5-point Likert scale from never to always. Three screening questions were included at the beginning of the questionnaire, to allow only people who have the intention to either have a healthy diet or not to eat much of foods they find tempting to answer the SREBQ. These screening questions were worded to fit both people who want to achieve a healthy diet and those who have achieved a healthy diet and want to maintain it. General terms such as *‘tempting foods’* were used throughout the questionnaire to enable people to respond to the questionnaire relative to their own eating intentions. The first pool of 102 items was reduced to 64 items after the first examination by the research team, based on the criteria of relevance, clarity and content.

#### Pilot study 1

The aim of this first pilot was to assess whether the items were easy to answer, unambiguous, and adequate and also to generate new items. This study was conducted with an opportunistic sample of 20 students and staff (60 % female) from University College London (UCL), aged 18 years or older (Sample 1). Participants answered the 64-item questionnaire alongside open and closed questions about whether they actually define eating goals for themselves and whether they can identify them and reflect on them. They were also invited to assess the items and make comments if they wanted. Open ended answers were entered into an Excel spreadsheet and analysed qualitatively. Answers to the open and closed questions around eating goals revealed that most participants reported defining their goals (85 %), but these goals varied in terms of level of abstraction, type of food and timeline. Items related to very specific goals were removed, for example *‘How often do you plan to bring a piece of fruit to work every day?’.* Other items were removed because they repeated the screening questions, (e.g. *‘how often do you set goals to eat healthily?*’), or were too similar to other questions. This resulted in the deletion of 22 items, generation of 15 new items and wording modifications to both the items and screening questions.

#### Pilot study 2

The aim of this second pilot was also to assess the adequacy of the items and to design new items. It used a larger and more varied convenience sample (Sample 2), and participants were recruited from two different sources. All members of the charity Weight Concern’s ‘Big Panel’ (an online panel of 1800 people who have a history of overweight or obesity), together with a wider sample of UCL staff and students were invited to participate via email. All participants were 18 years or over and no incentives were offered. The remaining 57 items were administered using an online survey platform (https://www.surveymonkey.com/). The survey was anonymous and participants were asked to answer the SREBQ and report their age, gender, weight and height. Open and closed questions were also included to assess participants’ eating goals, and perceptions of the relevance and adequacy of the items. Items which were positively and strongly correlated with BMI were also deleted as the SREBQ aims to assess eating self-regulatory skills associated with successful weight control. To ensure internal consistency of the item pool, items were culled when more than 60 % of the item-item correlation coefficients were lower than 0.3 [43], and when corrected item-total correlations were lower than 0.3 [44]. All psychometric and descriptive analyses were performed using IBM SPSS statistics version 22 (SPSS Inc., Chicago, IL, USA).

In total, 309 individuals accessed the questionnaire online, but only 193 adults completed the entire questionnaire and were included in the analyses. Of these, 77 % were women; 41.7 % were normal weight, 15.6 % were underweight, 17.7 % were overweight and 25 % were obese. The mean age was 40 years (SD 13.7). The majority of the participants (around 80 %) could identify eating goals they set for themselves. However, similar to the results from pilot study 1, participants’ goals varied in terms of level of abstraction, type of food and timeline. These results strengthened our decision to use general terms in the items, such as ‘*eating intentions’* and *‘tempting foods’*, as this allows people to relate items to their personal goals. Seventy one percent of the participants found the questionnaire easy and only 1 individual (0.5 %) found the questions offensive or displeasing. Around 60 % of the participants felt the questionnaire was assessing self-regulation of eating behaviour adequately. On the basis of the item-total correlation and item-item correlations and strong, positive associations with BMI, a total of 28 items were removed. The 29 items left were reworded and two new items were generated. For consistency all items using the term ‘*eating goals’* were reworded to *‘eating intentions’*. Additionally, an explanation was provided at the beginning of the questionnaire stating that *‘Eating intentions refer to the way you intend to eat (*e.g. *avoiding tempting foods and/or eating healthily)’*.

#### Internal reliability and initial factor structure study

The aim of this cross-sectional study was to investigate the underlying structure of the draft SREBQ and explore its internal reliability. Participants for this study were students and staff from UCL and members of 5 UK Facebook groups dedicated to discussion of weight loss and nutrition (Sample 3). Recruitment was via email and announcements posted on the groups’ Facebook pages, with potential participants provided with a link for online completion of the survey, comprising the 31-item SREBQ, and self-reported age, gender, weight and height. Participants were eligible for the study if they were aged 18 years or older; were living in the UK; had not taken part in the pilot studies and reported having eating intentions. All participants were invited to enter a prize draw for a £25 high street voucher. A total of 271 eligible participants completed the questionnaire and were included in the analysis. The majority were female (76.4 %) and the mean age was 31.5 years (SD 12). In terms of weight status, 8.4 % of the participants were underweight, 69.2 % were normal weight; 18.1 % overweight and 4.2 % were obese. Prior to factor structure analysis the scale was further refined in order to reduce item redundancy. Pairs of items with intra-item correlations greater than 0.6 [43] were identified and one of each pair of items was removed. The refinement criteria to choose one item in each pair were the same as those used in pilot study 2. The factor structure of the scale was determined by running Principal Component Analysis (PCA) with oblique rotation (since underlying components were expected to be correlated). Parallel analyses were also performed to help with the decision of the number of factors to retain. Factor loadings were expected to be greater than 0.4 [44]. To reduce participant burden and enhance the utility of the scale, the content and psychometrics of the retained items was reassessed, and items were removed where multiple items measured the same aspects of self-regulation. Following the refinement process, the PCA was re-examined. The Cronbach’s alpha for the final scale was calculated, which should be ≥0.7 [44]. All the psychometric and descriptive analyses were performed using IBM SPSS statistics version 22 (SPSS Inc., Chicago, IL, USA).

The initial refinement analyses removed 17 items. The PCA results for the 14 remaining items revealed a one-factor solution based on the Scree Plot and also on the parallel analysis (see Additional file 1). All items showed a factor loading greater than 0.4. However, content analyses of the remaining items indicated that there was still some redundancy and a total of 9 items were removed. The PCA was run a second time on the final 5-item questionnaire and produced a one-factor solution (see Additional file 2), accounting for 51.4 % of the variance (see Table 1).

**Table 1:** Factor structure of the 5-item SREBQ

Item	Factor loading	Capacity/ Processes
I’m good at resisting tempting food	.797	Ability to control behaviour, thoughts, feeling, attention and eat in accordance with your intentions/short-term capacity to regulate eating behaviours
I give up too easily on my eating intentions^R^	.789	Ability to stick to your eating intentions and continuously work toward them/long-term capacity to self-regulate eating behaviours
I easily get distracted from my eating intentions^R^	.746	Ability to control thoughts and attention and keep your eating goals in mind
I find it hard to remember what I have eaten throughout the day^R^	.618	Ability to monitor and be aware of your actual eating behaviour
If I am not eating in the way I intend to I make changes	.612	Ability to compare your actual behaviour to your eating intentions (reference) and make adjustments when necessary to achieve your intentions

Response scale for each item ranged from 1 (Never),to 5 (Always). ^R^Reverse item. Variance explained: 51.4 %. KMO = 0.80. Item-item correlation (range): 0.25 to 0.54. Item-total correlation (range):0.42 to 0.61

This final 5-item SREBQ included the main processes of self-regulation (self-monitoring, appraising progress, making amendments, giving up and overcoming barriers). The items also encompassed the capacity to control behaviour, thoughts and attention, supporting its content validity. The Cronbach’s alpha coefficient for the 5-item questionnaire was 0.75.

### Reliability and validity of the Self-regulation of Eating Behaviour Questionnaire study

This cross-sectional study aimed to assess the construct validity by confirming the final 5-item SREBQ’s structure, as well as the concurrent, convergent, and discriminant validities of the questionnaire. This study also aimed to assess the test-retest and confirm the questionnaire’s internal reliability.

#### Participants

The fourth sample was recruited through Research Now, an online market research company, which has access to a panel of over 6,000,000 UK residents and offers a small cash incentive for participation. A sample of 1000 is recommended as ideal for validation studies (Comrey and Lee, cited in [44]), so 1000 British adults aged between 20 to 65 years were recruited to the validation study and a second response obtained from 100 participants for the test-retest study. In order to obtain a more representative sample, the sample was stratified by gender (50 % Male); and weight status (55-60 % overweight or obese). Weight status percentages were established based on weight status statistics for the UK adult population [45]. To fulfil the required weight profile of the participants, age quotas were established based on the percentages of overweight and obese obtained per age group in a previous study conducted by our research group. This previous study collected data on eating behaviours and weight control from the Research Now Company using the same data collection techniques. Participants with a BMI lower than 14 kg/m^2^ or greater than 50 kg/m^2^ were excluded, as these values were considered too extreme and may represent unreliable self-reports of weight or height.

After quality checks, including time taken and pattern of responses, 46 responses were excluded. Thirty-one participants with missing data for the SREBQ were also omitted from the analysis, resulting in a final sample of 923 participants. For the test-retest 100 completed responses were obtained. The characteristics of the participants for both samples are presented in Table 2.

**Table 2:** Characteristics of the samples

	Total sample (*n* = 923)		Test-retest sample (*n* = 100)	
Variable	n	%	N	%
Gender				
Female	535	58	82	82
Male	388	42	18	18
Age				
20 to 29 years old	155	17	13	13
30 to 39 years old	167	18	17	17
40 to 49 years old	231	25	20	20
50 to 59 years old	238	26	24	24
60 to 65 years old	132	14	26	26
Ethnic group				
White	837	91	93	93
Black	20	2	1	1
Asian	40	4	3	3
Mixed	15	2	0	0
Other	11	1	3	3
Marital status				
Single	235	25	23	23
Married^a^	590	64	64	64
Separated/Widowed^b^	98	11	13	13
Education				
Primary/secondary school	79	9	13	13
O level to A levels^c^	289	31	37	37
Certificate/Diploma^d^	212	23	18	18
Degree^e^	343	37	32	32
Employment situation				
Paid work^f^	567	61	54	54
Unpaid work/unemployed^g^	210	23	24	24
Student	40	4	4	4
Retired	106	12	18	18
Living arrangement				
Own your home^h^	537	58	66	66
Renting^i^	312	34	30	30
Living with parents/University halls^j^	74	8	4	4
Weight status				
Underweight^k^	23	3	4	4
Normal weight^l^	363	39	43	43
Overweight^m^	273	30	24	24
Obese^n^	250	27	27	27
Missing^o^	14	1	2	2

^a^Married or living as married. ^b^Separated, divorced or widowed. ^c^O level/GCSEs/A levels. ^d^Technical or trade certificate/Diploma. ^e^Degree or Post-graduate degree. ^f^Employed full-time/Employed part-time/Self-employed ^g^Unemployed/Full-time homemaker/Unpaid or voluntary work/Disable or too ill to work. ^h^Own your home outright/ Own your home with mortgage. ^i^Rent from local authority or housing association/Rent privately. ^j^Living with parents/Living in University or College halls. ^k^BMI from 14 to 18.49 Kg/m^2^. ^l^BMI from 18.5 to 24.99 Kg/m^2^. ^m^BMI from 25 to 29.99 Kg/m^2^. ^n^BMI from 30 to 50 Kg/m^2^. ^o^Missing data includes: 2 participants with no data; 10 participants with BMI greater than 50 Kg/m^2^ and 2 participants with BMI lower than 14 Kg/m^2^

The final sample of 923 participants met the requirement of roughly equal numbers of male vs. female (42 % vs 58 %) and an age group balance. The sample also met the weight status requirement: 57 % of participants were overweight or obese and 39 % were of normal weight. The majority of participants were white (91 %), married (64 %); employed (61 %); and owned their own home (58 %). Around one third reported their highest education to be O levels to A levels (31 %), and just over one third had a degree (37 %). The test-retest sample was similar to the main sample, except for gender, where the majority of the participants for the test-retest were female (82 %).

#### Measures

The survey was administered using an online survey platform (https://www.surveymonkey.com/). Participants completed the 5-item SREBQ (see Additional file 3) and were asked to report their weight and height; gender; age; ethnicity; marital status; postcode; education; employment status and living arrangements. To assess dietary behaviours, participants answered four adapted food frequency questions, which were originally designed to be used with parents [46]. Respondents had to answer on a 7-point response scale that ranged from ‘less than once a week’ to ‘3 or more a day’ how frequently they eat fruits, vegetables, sweets and salty snacks, and sugary drinks.

In order to assess the concurrent validity of the questionnaire, participants had to answer questions from another 2 validated self-regulation questionnaires; the Perceived Self-Regulatory Success in Dieting Scale (PSRSDS) and the Brief Self-Control Scale (SCS). The PSRSDS is a 3-item questionnaire measuring how successful people are at dieting [47]. Participants rate on a 7-point scale how successful they are in watching their weight and losing weight, and also how difficult they find it to maintain their weight. The SCS is a 13-item scale measuring individuals differences in general self-control [19]. The scale was designed to assess the ability to break habits, resist temptations and maintain self-discipline. Participants were asked to answer on a 5-point response scale how well the items describe them.

Regarding the convergent validity of the SREBQ, participants were asked to answer other validated questionnaires for constructs likely to be related to eating self-regulatory skills. They answered the autonomous motivation subscale of the Dietary Self-Regulation Questionnaire, a 3-item sub-scale assessing the level of motivation to either start eating healthily or to continue to do so by rating on a 5-point scale their reasons for eating a healthy diet [48]. Participants also answered the Self-Report Habit Index, a 12-item scale [49], assessing the automaticity of avoiding tempting food on a 5-point response scale. Respondents also answered 2 subscales from the Adult Eating Behaviour Questionnaire (AEBQ), an adapted and validated version of the Child Eating Behaviour Questionnaire [50, 51], which measures a set of appetitive traits that confer risk of obesity. These were the four-item Food Responsiveness subscale, assessing interest in food and drive to eat, and the five-item Emotional Over-eating subscale, assessing the tendency to overeat in negative emotional states.

In order to assess the discriminant validity, participants were required to answer another 3 subscales from the AEBQ, which are related to better biological self-regulation, and therefore should not be related to intentional self-regulation. These were the 4-item Satiety Responsiveness subscale, measuring the individual’s sensitivity to fullness, the 5-item Food Fussiness subscale, and the 4-item Slowness in Eating subscale.

#### Procedure

This study received ethical approval from the University College London Ethics Committee (ID 5766/002). Panellists were invited via e-mail to complete the survey online. All participants gave informed consent. Only participants who intended to control their consumption of foods they find tempting or intended to have a healthy diet completed the SREBQ. The panellists who did not have any of these intentions were ineligible, as the items assume people have eating intentions. The questionnaire was found to take around 25 min and participants had one week to complete the questionnaire. Responses completed in 14 min or less were discarded, as this would not have allowed sufficient time for participants to read and complete the questionnaire. Questionnaires with the same answer for all items were also removed. To test the external (test-retest) reliability of the SREBQ, the first 200 respondents were re-contacted 2 weeks later and asked to complete the survey again. Two weeks is considered to be an acceptable length of time for participants not to be likely to remember their original responses exactly, nor to have had any notable changes in their level of self-regulation. Recruitment for the test-retest was closed when the required sample size of 100 was reached. First and second time responses were matched using panellists ID numbers.

#### Analysis

Having established the SREBQ’s single factor structure in the previous study, a Confirmatory Factor Analysis (CFA) was performed in order to confirm this structure. It is recommended to consult several goodness of fit statistics in order to assess whether the results are similar and judge if the model fits the data [52]. The indices most commonly used are the Chi square, which should be non-significant. However, Chi square very readily reaches significance with large sample sizes even when all other indices indicate a good fit [53]. Normed Fit Index (NFI) and Comparative Fit Index (CFI) should be close to 1 [53], which represent how much the model improves the fit relative to the null model. The Root-Mean-Square Error Approximation (RMSEA) represents a bad fit when greater than 0.1 [53].

As the data were normally distributed, Pearson’s correlations were used to assess the concurrent, convergent and discriminant validity. Regarding the concurrent validity, SREBQ scores were correlated to the scores for the SCS and the PSRSDS. With respect to the convergent validity, correlations between the scores for SREBQ and BMI [calculated by dividing individuals’ weight (kilograms) by the square of their height (metres)], consumption of fruit and vegetable; consumption of sweet and salty snacks; consumption of sugary drinks; autonomous motivation, automaticity, food responsiveness and emotional over-eating were conducted. Multiple regression analysis was performed to examine the independent contribution of each of these variables to the SREBQ score. In terms of the discriminant validity, correlations between the scores for the SREBQ and food fussiness, satiety responsiveness and slowness in eating were conducted. The SREBQ had its internal reliability examined, including the assessment of the corrected item-total correlation and the Cronbach’s alpha. Paired t-tests and Intra-class Correlation Coefficients (ICC) were calculated to assess the external reliability (test-retest). Minimum requirement for ICC is that it should be >0.7. All analyses were performed using IBM SPSS statistics version 22 (SPSS Inc., Chicago, IL, USA), except the CFA, which was performed using AMOS SPSS version 22 (SPSS Inc., Chicago, IL, USA). Descriptive analyses were done to characterize the samples Statistical significance was defined as a value of p ≤0.05.

## Results

**Fig. 2:**
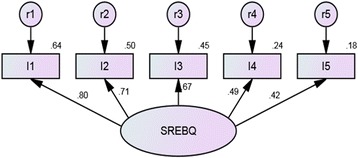
Final one-factor confirmatory factor analysis model for the SREBQ (*n* = 923). Note - Values over the arrow are the regression coefficient (Beta values). Values over the observed variables are the R^2^. I1 = I give up too easily on my eating intentions. I2 = I’m good at resisting tempting food. I3 = I easily get distracted from my eating intentions. I4 = If I am not eating in the way I intend to I make changes. I5 = I find it hard to remember what I have eaten throughout the day

Figure 2 shows the results for the CFA. The Chi square results were significant (*df* = 5; x^2^ = 29.400; *p* < 0.001). However, other model fit indices showed a good fit: NFI = 0.97; CFI = 0.97; TLI = 0.93 and RMSEA = 0.07. All the regression coefficients were greater than 0.4 and no modifications to the model were performed, demonstrating that the model fitted the data.

Correlations between the SREBQ and other measures of self-regulation are presented in Table 3. SREBQ score had a medium and positive correlation with the overall score for the PSRSDS and the SCS. In terms of the convergent validity, the SREBQ showed a small and positive correlation with fruit and vegetable intake; a small and negative correlation with sugary drinks consumption; and a medium and negative correlation with sweet and salty snack intake. These dietary variables showed a stronger correlation with SREBQ than with the other measures of self-regulation. In terms of weight status, SREBQ score had a small and negative correlation with BMI. This relationship was stronger than the correlation between SCS and BMI, but weaker than the correlation between PSSDS and BMI.

**Table 3:** Concurrent, Convergent and Discriminant validity tests of the SREBQ (*n* = 923)

Measure	1	2	3	4	5	6	7	8	9	10	11	12	13	14
Concurrent validity														
1. Self-Regulation of Eating Behaviour Questionnaire														
2. Perceived Self-Regulatory Success in Dieting Scale	.54**													
3. Self-Control Scale	.58**	.45**												
Convergent validity														
4. Fruit and vegetable consumption	.30**	.22**	.27**											
5. Sweet and salty snack consumption	−.40**	−.16**	−.26**	−0.02										
6. Sugary drinks consumption	−.23**	−.10**	−.21**	−.24**	.34**									
7. Body Mass Index	−.28**	−.55**	−.21**	−.09**	0.05	.07*								
8. Automaticity of avoiding tempting food	.60**	.46**	.41**	.30**	−.29**	−.17**	−.26**							
9. Motivation to have a healthy diet	.23**	.15**	.19**	.34**	−.07*	−.15**	−.10**	.21**						
10. Food Responsiveness	−.40**	−.21**	−.41**	−.06	.26**	.07*	.09**	−.18**	−.03					
11. Emotional overeating	−.40**	−.37*	−.40**	−.06	.20**	.12**	.28**	−.19**	−.07*	.43**				
Discriminant validity														
12. Food Fussiness	−.14**	−.10**	−.09**	−.18**	.12**	.19**	.04	−.09**	−.15**	−.10**	.08*			
13. Satiety Responsiveness	.062	.11**	.07*	−.08*	−.05	.08**	−.13**	.18**	−.05	−.23**	−.13**	.20**		
14. Slowness in eating	.07*	.14**	.09**	−.02	−.037	.05	−.10**	.09**	−.06	−.20**	−.13**	.06	.46**	

*p ≤ 0.05 (2-tailed) **p < 0.001 (2-tailed)

The SREBQ also showed a strong positive correlation with automaticity and also a positive, but small correlation with autonomous motivation to have a healthy diet. In addition, the results showed a medium and negative correlation with food responsiveness and emotional over-eating. In terms of the discriminant validity, the results showed a very small and negative correlation with food fussiness and a very small and positive correlation with satiety responsiveness and slowness in eating.

In order to see whether the convergent validity variables were independently associated with eating self-regulatory capacity, when adjusting for socio-demographic variables, a hierarchical multiple regression analysis was run (see Table 4). Variables entered at the first stage were age and gender, followed by weight and dietary variables and then by automaticity, motivation, food responsiveness and emotional overeating validity variables. The full model was statistically significant [*F*(10, 889) = 107.16, *p* < 0.001; R^2^ adjusted = .541] and accounted for 54.7 % of variance in SREBQ score. The addition of each block of independent variables led to a statistically significant increase in R^2^ (See Table 4). The results for the full model showed that higher SREBQ score was predicted by lower BMI; sugary drinks consumption; food responsiveness; and emotional over-eating, and by higher fruit and vegetable consumption; automaticity of avoiding tempting food; and motivation to have a healthy diet. Only sugary drinks consumption, was not independently related to eating self-regulatory capacity. Neither gender nor age significantly predicted eating self-regulatory capacity.

**Table 4:** Multiple regression analyses for the SREBQ

Model	Variables	SREBQ mean score (Full model)			R^2^ change	F statistic
		B	β	*p*		
1	Gender^a^	−.06	−.04	.052	.030	F(2897) = 13.6, *p* < 0.001
	Age	.00	−.00	.841		
2	Body Mass Index	−.01	−.08	<.001	.295	F(4893) = 97.6, *p* < 0.001
	Fruit and vegetable consumption	.05	.13	<.001		
	Sweet and salty snacks consumption	−.14	−.17	<.001		
	Sugary drinks consumption	−.03	−.03	.250		
3	Automaticity of avoiding tempting foods.	.36	.40	<.001	.222	F(889,4) = 108.6, *p* < 0.001
	Autonomous motivation to have a healthy diet	.05	.06	.013		
	Food responsiveness	−.16	−.19	<.001		
	Emotional over-eating	−.10	−.16	<.001		

Scores for self-regulation range from 1 to 5. ^a^Male = 0 and Female = 1. SREBQ constant: 3.0 (0.164)

The corrected item-total correlation of SREBQ ranged from 0.36 to 0.65, and the Cronbach’s alpha was 0.75. In terms of the test-retest results, the SREBQ showed an ICC of 0.77 (95%CI 0.67; 0.83) and the paired *t*-test was non-significant [t(99) = 0.59; *p* = 0.55].

## Discussion

The aim of the present study was to design and validate a measure to assess eating-related self-regulatory capacity for the UK adult population. The content of the SREBQ was informed by examining the literature and existing questionnaires on self-regulation; and by consulting experts in the field. The process of developing the SREBQ resulted in a 5-item questionnaire. The face validity was satisfactory and the factor structure analysis suggested that the questionnaire has one underlying factor. This structure was then tested in a different sample, and showed a good fit. Evidence for the construct validity of the SREBQ was demonstrated with tests of concurrent, convergent and discriminant validity.

Associations between the SREBQ and other measures of self-regulation were positive and represented a medium correlation, as expected [43]. The SREBQ was better at assessing self-regulatory capacity related to healthy diet than the SCS and PSRSDS. It was also better at assessing self-regulatory capacity related to weight control than the SCS. However, as expected, the PSRSDS showed a stronger correlation with BMI than the SREBQ, since the PSRSDS assesses self-regulatory capacity related specifically to weight control [47]. The SREBQ showed sufficient uniqueness in terms of non-shared variance and was better at assessing self-regulation of eating behaviour than existing measures. The SREBQ’s score was also associated with related constructs [6, 7, 30], such as automaticity, motivation for healthy diet, food responsiveness and emotional over-eating. Additionally, the SREBQ showed good discriminant validity, demonstrated by weak correlations with unrelated appetitive constructs [7], such as satiety responsiveness, food fussiness and slowness in eating.

The Multiple Regression model showed that the variables demonstrating convergent validity explained more than 50 % of the variance in the total score for the SREBQ. As anticipated, lower BMI, lower sweet and salty snacks consumption, and higher fruit and vegetable consumption significantly predicted eating self-regulatory capacity. The effect size was greater for sweet and salty snack consumption compared to the other diet variables. It has been suggested that ‘positive’ behaviours, such as the consumption of fruit and vegetables, more easily become habitual through routine and repetition of the behaviour, reducing the need for effortful self-regulation. On the other hand inhibiting ‘negative’ behaviours, such as avoidance of unhealthy foods, may require cognitively-mediated self-regulatory skills to be maintained [8, 54]. However, whether it is possible to form a habitual behaviour to avoid something should be further investigated by looking at the relationship between self-regulatory skills and automaticity of dietary behaviours longitudinally. Further studies are also needed to clarify why the relationship between self-regulation and sugary drinks consumption was not significant after adjusting for the other variables. We hypothesize that other factors, such as nutrition knowledge may play a moderator role in the relationship between self-regulation and sugary drinks consumption.

In the Multiple Regression model results for the related constructs, automaticity and motivation showed a positive and significant relationship with self-regulation capacity, while food responsiveness and emotional over-eating showed a significant negative relationship. The effect size was stronger for automaticity and weaker for motivation. These results seem to be supported by the literature. According to the COM-B system, in order to change a behaviour, sufficient motivation, capacity and opportunity are required [55]. The reflective motivation assessed in this study involves effortful behavioural processes [56], usually required during the process of behaviour change. Variance in reflective motivation resources may explain why some people experience self-regulatory failure during the behaviour change process [57]. As the individual achieves their intended behaviour, self-regulatory skills also becomes more automatic and efficient, requiring less reflective motivational resources [30, 54].

Finally, the regression results showed that eating self-regulatory skills were not related to age or gender. Some studies have shown that self-regulation may have an inverted U-shaped association with age [58, 59], increasing through adolescence and reducing in old age. The present study only included adults aged 20 to 65, and therefore no variation in self-regulation was expected. The gender results were also in accordance with the literature, as studies have shown that there are no significant differences in self-regulatory capacity between men and women over the life span [31, 42]. The five-item SREBQ also showed good internal and external reliability demonstrating that the questionnaire is measuring eating self-regulatory skills consistently and reproducibly.

There are some limitations that may affect the generalizability of these results. The findings regarding the validity and reliability are limited to the population of this study and the use of only self-report questionnaire measures. Future studies are needed to test the validity of the SREBQ in different populations (e.g. ethnic minorities and other countries) and against behavioural measures, and to explore the SREBQ’s predictive validity and responsiveness to change using longitudinal data. For convenience, university students and staff were invited to take part in the development process of the SREBQ and these are unlikely to reflect the educational and socio-economic status of the general population. However, the validity and reliability study included a more diverse sample of the UK population and found similar results. All data collection was online, which means that those without computer or internet access were excluded. There is also no information about how many people actually received the invitation but chose not to participate in each study. People with a greater interest in nutrition and weight control may have been more likely to have opted in. The results from the correlations and multiple regression analyses came from a cross-sectional study, and so cannot demonstrate causality. The self-report of weight and height may have introduced some inaccuracy to this data. However, studies have shown that adults, especially young adults, give a valid online self-report weight [60].

## Conclusions

The five-item Self-Regulation of Eating Behaviour Questionnaire is a novel measure of eating self-regulatory capacity that is consistent, reliable and valid for use in the general UK adult population. The validation process provided evidence that the SREBQ assesses people’s capacity to control and manage their eating behaviour in order to achieve and/or maintain their eating intentions. This new measure is likely to be useful for the assessment of the effectiveness of dietary and weight control interventions and particularly for assessing the effectiveness of interventions which aim to improve dietary self-regulation. Future studies should assess the relationships between self-regulation of eating behaviour, weight and diet using experimental and longitudinal study designs.

## Abbreviations

AEBQ, Adult Eating Behaviour Questionnaire; BMI, body mass index; CFA, Confirmatory Factor Analysis; CFI, Comparative Fit Index; NFI, Normed Fit Index; PSRSDS, Perceived Self-Regulatory Success in Dieting Scale; RMSEA, Root-Mean-Square Error Approximation; SCS, Brief Self-Control Scale; SREBQ, Self-Regulation of Eating Behaviour Questionnaire

## Additional files

Additional file 1: Scree plot and Parallel analyses of the 14 items retained in the ‘Internal Reliability and Factor Structure Study’. (DOCX 43 kb) Additional file 2: Scree plot and Parallel analyses of the final 5 items retained in the ‘Internal Reliability and Factor Structure Study’. (DOCX 38 kb) Additional file 3: Self-Regulation of Eating Behaviour Questionnaire. (DOCX 35 kb)

## References

[1] Malik VS, Willett WC, Hu FB (2013). Global obesity: trends, risk factors and policy implications. Nat Rev Endocrinol.

[2] Wardle J, Boniface D (2008). Changes in the distributions of body mass index and waist circumference in English adults, 1993/1994 to 2002/2003. Int J Obes (Lond).

[3] Johnson F, Pratt M, Wardle J (2012). Dietary restraint and self-regulation in eating behavior. Int J Obes (Lond).

[4] Kroese FM, Evers C, De Ridder DTD (2009). How chocolate keeps you slim. The effect of food temptations on weight watching goal importance, intentions, and eating behavior. Appetite.

[5] Hagger MS (2014). The multiple pathways by which trait self-control predicts health behavior. Ann Behav Med.

[6] Hofmann W, Schmeichel BJ, Baddeley AD (2012). Executive functions and self-regulation. Trends Cogn Sci.

[7] Llewellyn C, Wardle J (2015). Behavioral susceptibility to obesity: gene-environment interplay in the development of weight. Physiol Behav.

[8] Gardner B, Lally P, Wardle J (2012). Making health habitual: the psychology of ‘habit formation’ and general practice. Br J Gen Pract.

[9] Baumeister RF, Gailliot M, DeWall CN, Oaten M (2006). Self-regulation and personality: how interventions increase regulatory success, and how depletion moderates the effects of traits on behavior. J Pers.

[10] Boekaerts M, Maes S, Karoly P (2005). Self-regulation across domains of applied psychology: is there an emerging consensus?. Appl Psychol.

[11] Carver CS, Scheier MF (2001). On the self-regulation of behavior.

[12] De Vet E, De Ridder D, Stok M, et al. Assessing self-regulation strategies: development and validation of the tempest self-regulation questionnaire for eating (TESQ-E) in adolescents. Int J Behav Nutr Phys Act. 2014;11. 10.1186/s12966-014-0106-z PMC4161904 25231361

[13] Moilanen KL (2007). The adolescent self-regulatory inventory: the development and validation of a questionnaire of short-term and long-term self-regulation. J Youth Adolesc.

[14] de Ridder DT, Lensvelt-Mulders G, Finkenauer C, Stok FM, Baumeister RF (2012). Taking stock of self-control: a meta-analysis of how trait self-control relates to a wide range of behaviors. Personal Soc Psychol Rev.

[15] Gellert P, Ziegelmann JP, Lippke S, Schwarzer R (2012). Future time perspective and health behaviors: temporal framing of self-regulatory processes in physical exercise and dietary behaviors. Ann Behav Med.

[16] Allan JL, Johnston M, Campbell N (2011). Missed by an inch or a mile? Predicting the size of intention-behaviour gap from measures of executive control. Psychol Health.

[17] Bandura A (1991). Social cognitive theory of self-regulation. Organ Behav Hum Decis Process.

[18] Carver CS, Scheier MF (1982). Control-theory - a useful conceptual-framework for personality-social, clinical, and health psychology. Psychol Bull.

[19] Tangney JP, Baumeister RF, Boone AL (2004). High self-control predicts good adjustment, less pathology, better grades, and interpersonal success. J Pers.

[20] Stubbs RJ, Lavin JH (2013). The challenges of implementing behaviour changes that lead to sustained weight management. Nutr Bull.

[21] Dombrowski SU, Knittle K, Avenell A, Araujo-Soares V, Sniehotta FF. Long term maintenance of weight loss with non-surgical interventions in obese adults: systematic review and meta-analyses of randomised controlled trials. Br Med J. 2014;348. 10.1136/bmj.g2646 PMC4020585 25134100

[22] Kreausukon P, Gellert P, Lippke S, Schwarzer R (2012). Planning and self-efficacy can increase fruit and vegetable consumption: a randomized controlled trial. J Behav Med.

[23] Fishbach A, Friedman RS, Kruglanski AW (2003). Leading us not unto temptation: momentary allurements elicit overriding goal activation. J Pers Soc Psychol.

[24] Papies EK, Stroebe W, Aarts H (2009). Who likes it more? Restrained eaters’ implicit attitudes towards food. Appetite.

[25] Hankonen N, Kinnunen M, Absetz P, Jallinoja P (2014). Why do people high in self-control eat more healthily? Social cognitions as mediators. Ann Behav Med.

[26] Muraven M, Baumeister RF (2000). Self-regulation and depletion of limited resources: does self-control resemble a muscle?. Psychol Bull.

[27] Baumeister RF, Vohs KD, Tice DM (2007). The strength model of self-control. Curr Dir Psychol Sci.

[28] Carter EC, Kofler LM, Forster DE, McCullough ME. A Series of Meta-Analytic Tests of the Depletion Effect: Self-Control Does Not Seem to Rely on a Limited Resource. J Exp Psychol. 2015;144:1–20. 10.1037/xge0000083 26076043

[29] Converse PD, DeShon RP (2009). A tale of two tasks: reversing the self-regulatory resource depletion effect. J Appl Psychol.

[30] Bargh JA, Williams EL (2006). The automaticity of social life. Curr Dir Psychol Sci.

[31] Carey KB, Neal DJ, Collins SE (2004). A psychometric analysis of the self-regulation questionnaire. Addict Behav.

[32] Schroder KEE, Ollis CL, Davies S (2013). Habitual self-control: a brief measure of persistent goal pursuit. Eur J Personal.

[33] Mezo PG (2009). The Self-Control and Self-Management Scale (SCMS): development of an adaptive self-regulatory coping skills instrument. J Psychopathol Behav Assess.

[34] Kennett DJ, Nisbet C (1998). The influence of body mass index and learned resourcefulness skills on body image and lifestyle practises. Patient Educ Couns.

[35] Junger M, van Kampen M. Cognitive ability and self-control in relation to dietary habits, physical activity and bodyweight in adolescents. Int J Behav Nutr Phys Act. 2010;7. 10.1186/1479-5868-7-22 PMC2860342 20331887

[36] Herman CP, Mack D (1975). Restrained and unrestrained eating. J Pers.

[37] Williamson DA, Martin CK, York-Crowe E (2007). Measurement of dietary restraint: validity tests of four questionnaires. Appetite.

[38] Laessle RG, Tuschl RJ, Kotthaus BC, Pirke KM (1989). A comparison of the validity of three scales for the assessment of dietary restraint. J Abnorm Psychol.

[39] Schlundt DG, Zimering RT (1988). The Dieter’s inventory of eating temptations: a measure of weight control competence. Addict Behav.

[40] Nothwehr F, Dennis L, Wu HT (2007). Measurement of behavioral objectives for weight management. Health Educ Behav.

[41] Keller C, Siegrist M (2015). The weight management strategies inventory (WMSI). Development of a new measurement instrument, construct validation, and association with dieting success. Appetite.

[42] Kolodziejczyk JK, Norman GJ, Rock CL, et al. Reliability and concurrent and construct validity of the strategies for weight management measure for adults. Obes Res Clin Pract. 2015;10:291–303. 10.1016/j.orcp.2015.06.004 PMC4729664 26227996

[43] Streiner DL, Norman GR (2008). Health measurement scales: a practical guide to their development and use Oxford University Press.

[44] Field A (2013). Discovering Statistics using IBM SPSS Statistics.

[45] Health Survey of England. Chapter 10: Adult antropometric measures, overweight and obesity. London: Health and Social Care Information Centre; 2012.

[46] Croker H, Lucas R, Wardle J (2012). Cluster-randomised trial to evaluate the ‘Change for Life’ mass media/social marketing campaign in the UK. BMC Public Health.

[47] Meule A, Papies EK, Kubler A (2012). Differentiating between successful and unsuccessful dieters. Validity and reliability of the Perceived Self-Regulatory Success in Dieting Scale. Appetite.

[48] Levesque CS, Williams GC, Elliot D (2007). Validating the theoretical structure of the Treatment Self-Regulation Questionnaire (TSRQ) across three different health behaviors. Health Educ Res.

[49] Verplanken B, Orbell S (2003). Reflections on past behavior: A self-report index of habit strength. J Appl Soc Psychol.

[50] Wardle J, Guthrie CA, Sanderson S, Rapoport L (2001). Development of the children’s eating behaviour questionnaire. J Child Psychol Psychiatry.

[51] Hunot C, Fildes A, Croker H (2016). Appetitive traits and relationships with BMI in adults: Development of the Adult Eating Behaviour Questionnaire. Appetite.

[52] Thompson B. Exploratory and confirmatory factor analysis: understanding concepts and applications (reprint Ed.). Washington: American Psychological Association; 2004.

[53] Dugard P, Todman J, Staines H, Dugard P, Todman J, Staines H (2010). Factor analysis. Approaching multivariate analysis: a practical introduction.

[54] Marteau TM, Hollands GJ, Fletcher PC (2012). Changing human behavior to prevent disease: the importance of targeting automatic processes. Science.

[55] Michie S, van Stralen MM, West R (2011). The behaviour change wheel: a new method for characterising and designing behaviour change interventions. Implement Sci.

[56] Bandura A (2005). The primacy of self-regulation in health promotion. Appl Psychol.

[57] Muraven M, Slessareva E (2003). Mechanisms of self-control failure: motivation and limited resources. Personal Soc Psychol Bull.

[58] Williams BR, Ponesse JS, Schachar RJ, Logan GD, Tannock R (1999). Development of inhibitory control across the life span. Dev Psychol.

[59] Hippel WV, Henry JD, Vohs KD, Baumeister RF (2011). Aging and self-regulation. Handbook of self-regulation: research, theory and applications.

[60] Pursey K, Burrows TL, Stanwell P, Collins CE (2014). How accurate is web-based self-reported height, weight, and body mass index in young adults?. J Med Internet Res.

