# Supplementary material for: Development and validation of the Self-Regulation of Eating Behaviour Questionnaire for adults
Source: Int J Behav Nutr Phys Act. 2016 Aug 2;13:87. doi: 10.1186/s12966-016-0414-6 (PMC4969721; doi:10.1186/s12966-016-0414-6)

**Additional file 2**: Scree plot and Parallel analyses of the final 5 items retained in the ‘Internal Reliability and Factor Structure Study’

a) Scree Plot


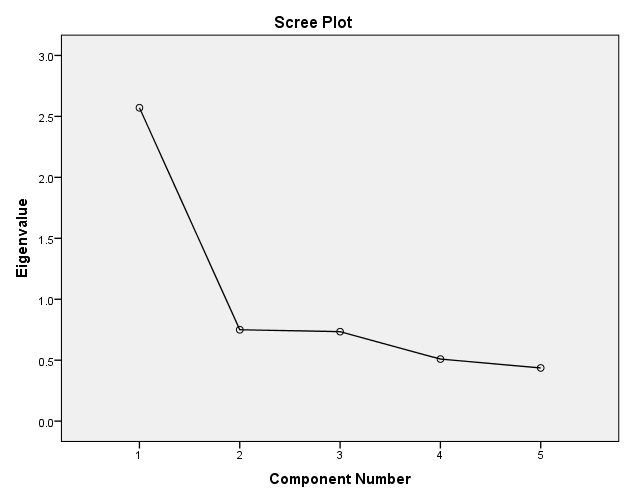


b) Parallel Analyses


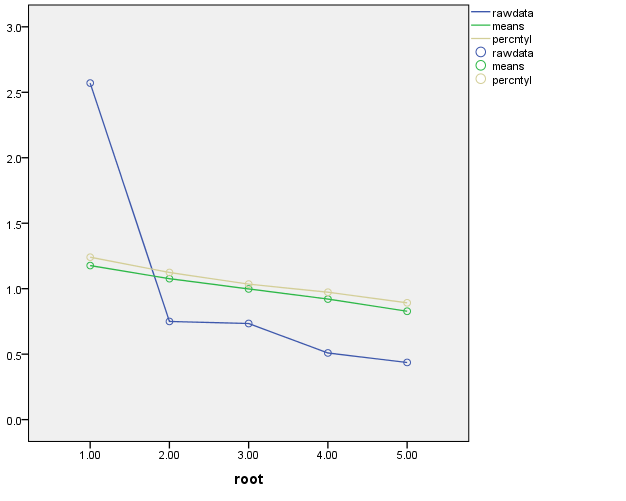

Supplement: Additional file 2: — Scree plot and Parallel analyses of the final 5 items retained in the ‘Internal Reliability and Factor Structure Study’. (DOCX 38 kb) [file 12966_2016_414_MOESM2_ESM.docx]
