# Supplementary material for: Development and validation of the Self-Regulation of Eating Behaviour Questionnaire for adults
Source: Int J Behav Nutr Phys Act. 2016 Aug 2;13:87. doi: 10.1186/s12966-016-0414-6 (PMC4969721; doi:10.1186/s12966-016-0414-6)
Supplement: Additional file 3: — Self-Regulation of Eating Behaviour Questionnaire. (DOCX 35 kb) [file 12966_2016_414_MOESM3_ESM.docx]

## Additional file 3: Self-Regulation of Eating Behaviour Questionnaire

**Screening questions:**

**1.** **Do you find any of these foods tempting (that is, do you want to eat more of them than you think you should)? (Tick those which apply)**

| □ | Chocolate | □ | Fizzy drinks | □ | Pizza |
| --- | --- | --- | --- | --- | --- |
| □ | Crisps | □ | Biscuits | □ | Fried foods |
| □ | Cakes | □ | Sweets | □ | Chips |
| □ | Ice cream | □ | Popcorn | □ | Other foods |
| □ | Bread/toast | □ | Pastries | □ | I don’t find any food tempting |

If you have ticked other foods, please specify:

|  |
| --- |

**2. Do you intend NOT to eat too much of these foods you find tempting in the previous question?**

| □ | Yes |
| --- | --- |
| □ | No |

**3. Do you intend to have a healthy diet?**

| □ | Yes |
| --- | --- |
| □ | No |

**Self-Regulation of Eating Behaviour Questions:**

**4.** **Please read the following statements and tick the boxes most appropriate to you.**

For the next few questions, please, understand that:

- ‘Tempting foods’ are any food you want to eat more of than you think your should.

- ‘Eating intentions’ refer to the way you are aiming to eat, for example you may intend to avoid tempting foods or eat healthy foods.

|  | | **Never** | **Rarely** | **Sometimes** | **Often** | **Always** |
| --- | --- | --- | --- | --- | --- | --- |
| 1 | I give up too easily on my eating intentions | □ | □ | □ | □ | □ |
| 2 | I'm good at resisting tempting food | □ | □ | □ | □ | □ |
| 3 | I easily get distracted from the way I intend to eat | □ | □ | □ | □ | □ |
| 4 | If I am not eating in the way I intend to I make changes | □ | □ | □ | □ | □ |
| 5 | I find it hard to remember what I have eaten throughout the day | □ | □ | □ | □ | □ |

## Total score (Mean) cut-off points:

| <2.8 | Low |
| --- | --- |
| 2.8 to 3.6 | Medium |
| >3.6 | High |
